# Citric acid disassembles α-synuclein fibrils and reduces their cytotoxicity

**DOI:** 10.1016/j.jpha.2025.101404

**Published:** 2025-07-10

**Authors:** Hyo Gi Jung, Yeon Ho Kim, Junho Bang, Dongsung Park, Jae Won Jang, Yonghwan Kim, Hyunji Kim, Kyo Seon Hwang, Jeong Hoon Lee, Dongtak Lee, Dae Sung Yoon

**Affiliations:** a School of Biomedical Engineering, Korea University, Seoul, 02841, Republic of Korea; b Interdisciplinary Program in Precision Public Health, Korea University, Seoul, 02841, Republic of Korea; c Department of Clinical Pharmacology and Therapeutics, College of Medicine, Kyung Hee University, Seoul, 02447, Republic of Korea; d Korea University-Korea Institute of Science and Technology (KU-KIST) Graduate School of Converging Science and Technology, Korea University, Seoul, 02841, Republic of Korea; e Department of Integrative Energy Engineering, College of Engineering, Korea University, Seoul, 02841, Republic of Korea; f Department of Bioengineering & Nano-bioengineering, Research Center for Bio Materials and Process Development, Incheon National University, Incheon, 22012, Republic of Korea; g Division of Bioengineering, Incheon National University, Incheon, 22012, Republic of Korea; h Astrion Inc., Seoul, 02841, Republic of Korea

## Abstract

• Fibrillar aggregates of α‑synuclein induce neuronal degeneration in the brain.
• Citric acid can disrupt the β-sheet structure of α-synuclein fibrils and inhibit additional fibril formation.
• The molecular docking simulation shows that citric acid exhibits a strong affinity for β-sheet stacking and adjacent regions.
• Citric acid reduces the cytotoxicity of α-synuclein fibrils.

## Graphical abstract

**Figure:**
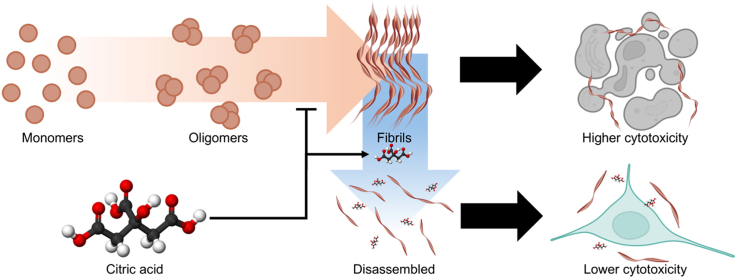

α-synuclein plays a crucial role in regulating neurotransmitter release and synaptic plasticity [1]. However, misfolding and aggregation of α-synuclein are major hallmarks of Lewy bodies related to progressive neurodegenerative disorders, including Parkinson’s disease, dementia with Lewy bodies, and multiple system atrophy [2]. Thus, developing therapeutics that target α-synuclein aggregates is crucial for treating synucleinopathies, as it alleviates neurotoxicity [3]. Herein, we demonstrate that citric acid (citrate; 2-hydroxy-propane-1,2,3-tricarboxylic acid), commonly found in food supplements, can disassemble α-synuclein fibrils and mitigate their neuronal toxicity (Fig. 1A). Our findings reveal that citric acid can effectively disrupts β-sheet structure of α-synuclein fibrils and inhibit further fibril formation. Molecular docking (MD) simulation further supports these results, showing that citric acid exhibits a high affinity for the β-strand and the surrounding region within α-synuclein fibrils. We propose that citric acid holds significant promise for pharmaceutical applications in treating neurodegenerative diseases related to synucleinopathies.

**Fig. 1:**
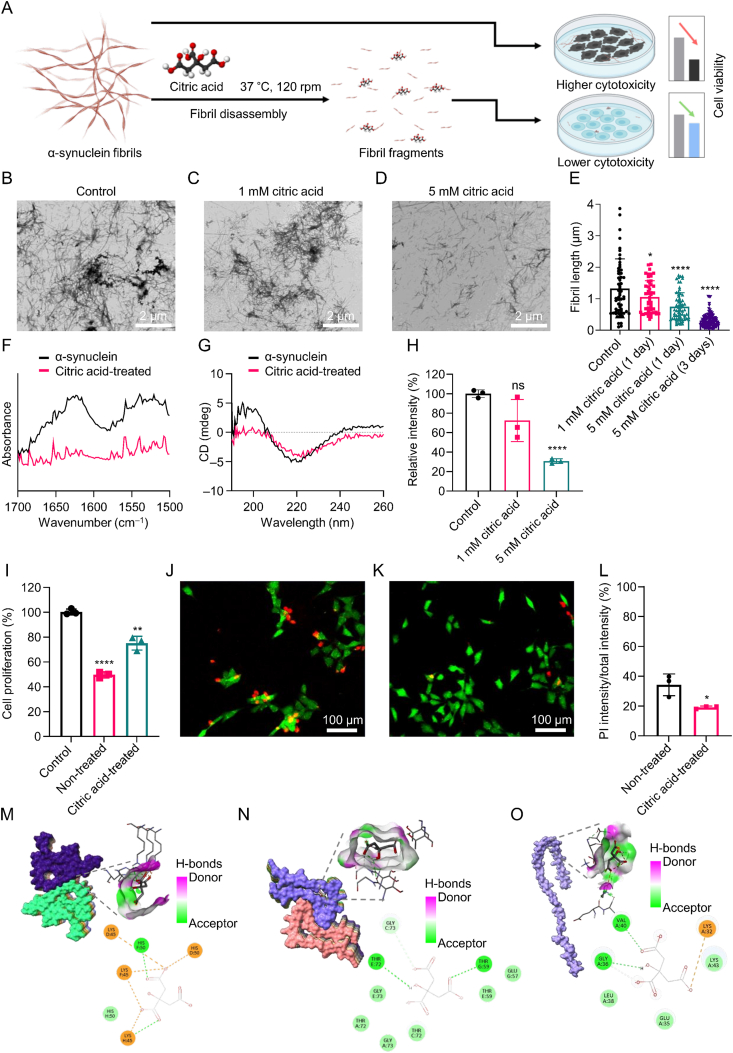
Physicochemical and computational analysis of citric acid-induced disassembly of α-synuclein fibrils and alleviation of their cytotoxicity. (A) The schematic illustration of the drug action of citric acid on α-synuclein fibrils. Citric acid can disassemble fibrillar α-synuclein aggregates and reduce the cytotoxicity of α-synuclein fibrils. (B–D) The transmission electron microscopy (TEM) images of α-synuclein fibrils (B), and α-synuclein fibrils reacted with 1 mM (C) and 5 mM (D) of citric acid for one day. (E) The average length of α-synuclein fibrils treated with 1 mM of citric acid for one day, 5 mM of citric acid for one day, and 5 mM of citric acid for three days. The lengths of each sample are measured using the ImageJ software. Data are presented as the mean ± standard deviation (SD) (*n* = 60 for the setup with only fibrils, *n* = 52 for the setup with 1 mM of citric acid (one day), *n* = 72 for the setup with 5 mM of citric acid (one day), and *n* = 101 for the setup with 5 mM of citric acid (three days)). (F) The Fourier-transform infrared (FT-IR) spectra of α-synuclein fibrils (black) and citric acid-treated α-synuclein fibrils (red). (G) The circular dichroism (CD) spectra of α-synuclein fibrils (black) and citric acid-treated α-synuclein fibrils (red). (H) The thioflavin T (ThT) intensity of α-synuclein fibrils reacted with various concentrations of citric acid (0, 1, and 5 mM). Data are presented as the mean ± SD (*n* = 3 independent experiments). (I) Cytotoxicity of α-synuclein fibrils and citric acid-treated α-synuclein fibrils in HT-22 cells. Data are presented as the mean values ± SD (*n* = 3 independent experiments). (J, K) Confocal fluorescent images of HT-22 cells incubated with α-synuclein fibrils (J) and citric acid-treated α-synuclein fibrils (K). Each cell is reacted with a live/dead staining kit. Dead cells are stained with propidium iodide (red) and living cells are stained with calcein-acetoxymethyl ester (AM) (green). (L) The relative percentage of propidium iodide fluorescence intensity to total fluorescence intensity for fibrils with/without citric acid treatment. Data are presented as the mean ± SD (*n* = 3 independent experiments). (M–O) Molecular docking (MD) simulation (AutoDock Vina) of α-synuclein fibrils and citric acid (PubChem CID: 311) at pH 7.2: rod polymorph form of the α-synuclein fibril (Protein Data Bank (PDB) ID: 6CU7) (M), twist polymorph form of the α-synuclein fibril (PDB ID: 6CU8) (N), and MD simulation (AutoDock Vina) of an α-synuclein monomer (PDB: 1XQ8) and citric acid (PubChem CID: 311) (O). ∗*P* < 0.05, ^∗∗^*P* < 0.01, and ^∗∗∗∗^*P* < 0.0001. ns: not siginificant.

To investigate the inhibitory effect of citric acid on α-synuclein aggregation, we conducted thioflavin T (ThT) assays (Fig. S1). The result showed that citric acid suppressed aggregation by interfering with β-sheet formation. Subsequently, α-synuclein fibrils were fabricated (Fig. S2) and treated with varying citric acid concentrations (Figs. 1B−D). These results are quantitatively analyzed revealing that the average length of α-synuclein fibrils decreased from 1.33 ± 0.93 μm at 0 mM to 1.06 ± 0.51 μm at 1 mM and further to 0.79 ± 0.55 μm at 5 mM (Fig. 1E). It indicates a significant reduction in both the number and average length of α-synuclein fibrils as the citric acid concentration increases. This demonstrates the efficient disassembly of the fibrillar α-synuclein aggregates by citric acid. In addition, a notable reduction in fibril length was observed with prolonged incubation time (Fig. S3). The ability of citric acid to disassemble α-synuclein fibrils was further validated by immunoblot analysis (Fig. S4) and atomic force microscopy (AFM) analysis (Fig. S5).

To investigate how citric acid disassembles α-synuclein fibrils, we analyzed the conformational changes using the Fourier-transform infrared (FT-IR), circular dichroism (CD), and ThT assays (Figs. 1F–H). The FT-IR spectrum between 1700 and 1500 cm^−1^ represents the Amide I′ and Amide II' bands, which are used to monitor the conformation changes in protein structure. The CD spectra exhibited distinct peaks between 190–200 nm and 220–230 nm, indicating the β-sheet structures in fibrils. These peaks were significantly reduced when α-synuclein fibrils were incubated with 5 mM citric acid, validating its ability to disassemble the β-sheet structure of α-synuclein fibrils. In addition, the ThT fluorescence signal of α-synuclein fibrils treated with citric acid (1 and 5 mM) exhibited a decrease in fluorescence intensity by 28% (1 mM) and 70% (5 mM), in comparison to that of the control (0 mM). The reduction of fluorescence signal directly indicates a corresponding decrease in the amount of β-sheet structures in the amyloid fibrils due to citric acid. The ThT assay was also conducted in artificial cerebrospinal fluid (aCSF) and neutral pH (Fig. S6), indicating that citric acid remains effective under aCSF and neutral pH conditions. These propensities of citric acid to break down the β-strand structure of amyloid fibrils are comparable to previous studies on α-synuclein fibril-disassembling small molecules [3].

To determine a suitable citric acid concentration for cellular assays, we assessed cytotoxicity using neuronal cell lines at various concentrations (Fig. S7) and selected 5 mM for further experiments. Subsequently, α-synuclein fibrils treated with citric acid were added to the neuronal cells to observe the therapeutic effecacy. Fig. 1I shows that 11 μM of α-synuclein fibrils decreased by 50.4% cell viability and citric acid treated α-synuclein fibrils decreased by 24.9% cell viability. This result indicates that citric acid mitigated the cytotoxicity of α-synuclein fibrils by 51% and also suggesting its potential neuroprotective effect of citric acid against α-synuclein fibrils. The confocal fluorescence microscopy image revealed that citric acid significantly reduced the number of dead cells relative to the untreated group (Figs. 1J and K). These results were quantitatively analyzed using the ratio of the cell death area in Fig. 1L, which shows that citric acid reduced the dead cell ratio of α-synuclein fibrils from 34.24% ± 5.9% to 19.05% ± 0.76%. Collectively, these results indicate that citric acid alleviated the cytotoxicity of α-synuclein fibrils, suggesting its potential as a drug candidate for synucleinopathies.

To understand the interaction between citric acid and α-synuclein fibrils, we performed a MD simulation at pH 7.2 (Figs. 1M and N; Tables S1 and S2) and at pH 2.98 (Figs. S8 and S9; Tables S3 and S4). The results showed that citric acid has a high affinity for α-synuclein fibrils at a β-strand formation region (HIS50, and THR72) and the vicinity of the β-strand formation region (LYS45, THR59, and GLY73) within α-synuclein sequences [4] (Tables S1−S4). We also conducted an MD simulation involving citric acid and an α-synuclein monomer (Fig. 1O). The results revealed interactions between citric acid and the β-strand formation region, as well as the vicinity of the β-strand formation region (GLY36 and VAL40), of α-synuclein monomer [4] (Table S5). Both MD results of citric acid on α-synuclein fibril and monomer suggest that the strong affinity of citric acid for β-sheet forming regions and their surrounding areas of α-synuclein fibrils leads to β-stack disassembly and inhibition in the fibril formation. All detailed methods and materials are shown in Supplementary data.

In summary, we have demonstrated that citric acid can effectively inhibits the α-synuclein fibril formation and disaggregates existing fibrillar aggregates. It also reduces the cytotoxicity of α-synuclein fibrils and interacts with the β-strand regions and their surrounding residues within the fibril structure. Given that pathological α-synuclein aggregates may originate in the gut, orally administered citric acid could act locally to disassemble them before brain propagation. Notably, although typical plasma citrate levels are around 0.1 mM, gastrointestinal concentrations can transiently reach 10–20 mM after citrus ingestion, supporting the feasibility of our effective dose for gut-targeted intervention.

## CRediT authorship contribution statement

**Hyo Gi Jung:** Writing – review & editing, Writing – original draft, Investigation, Funding acquisition, Formal analysis, Conceptualization. **Yeon Ho Kim:** Writing – original draft, Investigation, Formal analysis, Data curation, Conceptualization. **Junho Bang:** Investigation, Data curation. **Dongsung Park:** Formal analysis. **Jae Won Jang:** Funding acquisition, Data curation. **Yonghwan Kim:** Data curation. **Hyunji Kim:** Investigation. **Kyo Seon Hwang:** Supervision. **Jeong Hoon Lee:** Writing – review & editing, Supervision. **Dongtak Lee:** Writing – review & editing, Writing – original draft Conceptualization, Validation, Supervision, Funding acquisition. **Dae Sung Yoon:** Writing – review & editing, Writing – original draft, Validation, Supervision, Funding acquisition, Conceptualization.

## Declaration of competing interest

The authors declare that they have no known competing financial interests or personal relationships that could have appeared to influence the work reported in this paper.

## References

[1] Wenning G.K., Jellinger K.A. (2005). The role of α-synuclein in the pathogenesis of multiple system atrophy. Acta Neuropathol.

[2] Surguchov A., Surguchev A. (2022). Synucleins: New data on misfolding, aggregation and role in diseases. Biomedicines.

[3] Murray K.A., Hu C.J., Pan H. (2023). Small molecules disaggregate alpha-synuclein and prevent seeding from patient brain-derived fibrils. Proc. Natl. Acad. Sci. U S A.

[4] Guerrero-Ferreira R., Taylor N.M., Arteni A.-A. (2019). Two new polymorphic structures of human full-length alpha-synuclein fibrils solved by cryo-electron microscopy. Elife.

