# Supplementary material for: Citric acid disassembles α-synuclein fibrils and reduces their cytotoxicity
Source: J Pharm Anal. 2025 Jul 10;16(4):101404. doi: 10.1016/j.jpha.2025.101404 (PMC13091190; doi:10.1016/j.jpha.2025.101404)
Supplement: Multimedia component 1 [file mmc1.docx]

**Supporting Information**

**Citric Acid Disassembles α-Synuclein Fibrils and Reduces Their Cytotoxicity**

1. **Introduction**

α-synuclein, a soluble cytoplasmic protein, plays a pivotal role in regulating neurotransmitter release and synaptic plasticity in the presynaptic terminal of neurons [1]. In addition, recent studies have revealed that α-synuclein exhibits chaperone-like activity and controls dopamine release and the presynaptic soluble N-ethylmaleimide-sensitive-factor attachment protein receptor (SNARE) protein complex [2, 3]. However, the misfolding and aggregation of α-synuclein are associated with the progression of various neurodegenerative diseases, including Parkinson’s disease (PD), multiple system atrophy, and dementia with Lewy bodies (DLB) [4, 5]. In particular, fibrillar aggregates of α-synuclein constitute the major component of Lewy bodies, causing neuronal degeneration in the substantia nigra region of patients with PD [6].

The fibrillization of α-synuclein is affected by the multiplication of the *SNCA* gene and mutations of α-synuclein (e.g., E46K, A53T, and A30P) [7]. Environmental factors, including dopamine, metal ions, and pesticides, accelerate α-synuclein oligomerization and fibrillization [8-11]. In addition, the structure-forming effects of low pH and high temperature contributed to the formation of α-synuclein fibrillar aggregates [12, 13]. These fibrillar aggregates can secrete soluble α-synuclein oligomers, which can spread to adjacent cells and cause secondary nucleation sites with neural toxicity [14, 15]. Although various strategies, including small molecules [16-18], antibodies [19, 20], and engineered peptides [21-23], can effectively prevent the fibrillar aggregation of α-synuclein, existing aggregates remain active in the brain and can induce additional spread of α-synuclein aggregates [24, 25]. Therefore, the most desirable treatment against synucleinopathies would involve disassembling and removing the existing fibrillar form of α-synuclein and preventing further α-synuclein aggregation in the brain.

Citric acid (citrate, 2-hydroxy-propane-1,2,3-tricarboxylic acid), an intermediate product of the tricarboxylic acid cycle, is an organic acid that is widely used across industries such as foods, cosmetics, and pharmaceuticals [26, 27]. Numerous studies have highlighted the ability of citric acid to reduce inflammation and suppress lipid peroxidation by regulating the release of key inflammatory markers, including interleukin-1β, elastase, tumor necrosis factor-α, and myeloperoxidase [28-30]. In this regard, citric acid attenuates the oxidative stress of the brain [28, 31] and exhibits a beneficial impact on neuronal injuries [31]. To further explore its beneficial effects on the brain, we conducted an in-depth exploration of its intervention effects on α-synuclein fibrillar aggregates. Our analysis was performed using both atomic force microscopy (AFM) and transmission electron microscopy (TEM), revealing that citric acid efficiently breaks down α-synuclein fibrils. We corroborated this finding by assessing the conformational changes in α-synuclein fibrils using Thioflavin T (ThT), circular dichroism (CD), and Fourier-transform infrared (FT-IR) assays. The molecular docking (MD) simulation further supported our results, indicating that citric acid exhibits a strong affinity for β-sheet stacking and adjacent regions. Notably, citric acid significantly reduces the cytotoxicity associated with α-synuclein fibrils. Altogether, we propose the potential of citric acid as an α-synuclein aggregate-targeting agent for synucleinopathy-related diseases.

1. **Materials and methods**
   1. *Materials*

Lyophilized α-synuclein monomer powder was obtained from rPeptide (USA). ThT, citric acid (ACS reagent, ≥ 99.5%), and Dulbecco’s Modified Eagle’s Medium–high glucose (DMEM-h) were sourced from Sigma-Aldrich (USA). The Live/Dead cell staining kit was procured from Biomax (Republic of Korea). Distilled water (DW) and 1× phosphate-buffered saline (PBS) (pH 7.4) were purchased from Gibco (USA). The HT-22 cell line was obtained from the Korean Cell Line Bank (Republic of Korea) and used at passages no greater than 9. Cell Counting Kit-8 (CCK-8) was procured from Dojindo Laboratories (Republic of Korea). α-synuclein monoclonal antibody (Syn 211, Catalog # 32-8100, Lot # 3425410) was purchased from Invitrogen (USA). Tris-Glycine eXtended (TGX) FastCast Acrylamide Solutions were obtained from Bio-Rad (USA).

- 1. *Preparation of α-synuclein fibrils and citric acid treatment*

Before fabricating the α-synuclein fibrils, lyophilized α-synuclein monomer powder was dissolved in 1× PBS to a final concentration of 1 mg·mL^-1^ and aliquoted into 1.7-mL microtubes. The aliquoted α-synuclein solutions were incubated at 60℃ with shaking at 1,000 revolutions per min (RPM). in a thermomixer (Eppendorf, Germany) for five days to promote α-synuclein fibril formation. Afterward, fabricated fibrils were confirmed by AFM imaging and stored at 4 ℃ in a refrigerator. To observe the reaction of citric acid to α-synuclein fibrils, citric acid was added to the α-synuclein fibril solution to achieve the final concentrations of 1 and 5 mM. Then, the mixtures were incubated for one to three days at 37℃ to allow citric acid to treat α-synuclein fibrils.

- 1. *AFM imaging*

Before the sample deposition, a silicon wafer was washed with a piranha solution, which was prepared by mixing H_2_SO_4_ and H_2_O_2_ in a 1:1 ratio. Afterward, a 20-μL aliquot of α-synuclein fibril solution was placed on the silicon wafer at room temperature within a fume hood for 20 min. The silicon wafer with the deposited sample was then washed with DW and dried using N_2_ gas for 5 min. AFM analysis was performed using an NX10 instrument (Park Systems, Republic of Korea) equipped with a silicon tip (NCHR, Park Systems, Republic of Korea)[32]. Sample measurements were conducted in a non-contact mode which has a scanning rate of 0.5 Hz. The image is 5μm × 5μm in size.

- 1. *TEM analysis*

Before the analysis, fibril samples, with or without citric acid treatment, were diluted 10-fold with DW. The samples were fixed for 12 h in a solution containing 2% glutaraldehyde and 2% paraformaldehyde in 0.1 M phosphate buffer at pH 7.4, then washed with 0.1 M phosphate buffer. The samples were then post-fixed with 1% OsO_4_ in 0.1 M phosphate buffer for 2 h and dehydrated through a series of ethanol solutions for 10 min each. The concentrations of ethanol solution were 50, 60, 70, 80, 90, 95, and twice at 100%. The samples were then infiltrated with propylene oxide for 10 min. The samples were embedded using a Poly/Bed 812 kit from Polysciences (USA) and polymerized in a TD-700 electron microscope oven (DOSAKA, Japan) at 65℃ for 12 h. The block was cut into semi-thin sections of 200 nm thickness using a diamond knife in the Ultramicrotome and stained with toluidine blue for observation under an optical microscope. The region of interest was then cut into thin sections of 80-nm thickness using an ultramicrotome and placed on copper grids. The sections were double-stained with 3% uranyl acetate for 30 min and 3% lead citrate for 7 min before being imaged at an acceleration voltage of 80 kV. The microscope was equipped with a Megaview III CCD camera (Soft Imaging System, Germany), and TEM images were obtained using a JEM-1011 microscope (JEOL, Japan). The average length and standard deviation of each fibril sample were measured and calculated using the ImageJ software.

- 1. *Slot blotting of fibril samples*

Before preparing citric acid-treated α-synuclein fibril samples, the fibril solution was initially centrifuged at 25,000 × g for 1 h at 4℃. The supernatant was subsequently removed to ensure only α-synuclein fibrils remained in the solution. The citric acid treated, and non-treated fibril samples are loaded on polyvinylidene fluoride (PVDF) membrane and dried for 90 min at room temperature. The membrane was blocked with non-fat dry milk in PBS/0.1% Tween 20 for 30 min. Each membrane was incubated with rabbit OC (ARIGO, Taiwan) for 45 min. The membrane was washed three times in PBS/0.1% Tween 20 and incubated with Horseradish Peroxidase (HRP)-conjugated anti-rabbit IgG (ABclonal, USA). The sample visualization was conducted by Pico solution (luminol-based peroxidase substrate (LPS) solution). The resulting image was obtained using the iBright™ CL750 Imaging System (Invitrogen, USA).

- 1. *FT-IR measurements*

Before the analysis, 20 μL of both the α-synuclein fibril and citric acid-treated α-synuclein fibril solutions were deposited onto a silicon wafer that had been rinsed with piranha solution. Deposited samples were dried using N_2_ gas for 5 min before the FT-IR spectra measurement. FT-IR data were collected using a Cary 630 FT-IR instrument (Agilent, USA) and the spectral resolution was set to 4 nm [33]. The scanning range was 1600–1700 cm^-1^ in wavenumber.

- 1. *CD measurements*

A 50-μL aliquot of both the α-synuclein fibril and the citric acid-treated α-synuclein fibril solutions was placed into a quartz glass cuvette (Aireka Cells, USA) with a path length of 1 mm and an inside width of 10 mm. Circular dichroism spectra of each sample were obtained using a J-1100 spectrophotometer (JASCO, Japan) [33]. The spectra were measured a range of 190–300 nm at a scanning rate of 10 nm·min^-1^ and a resolution of 8 nm.

- 1. *ThT fluorescence assay*

ThT was dissolved in DW and then filtered using a 0.22-µm filter to make a final concentration of 1 mM. This stock solution was diluted to a concentration of 5 µM by adding 5 µL of the stock solution to 995 µL of DW. Afterward, 30 µL of α-synuclein fibril solution was incubated with 70 µL of the 5 µM ThT solution for 5 min. The fluorescence intensity was measured using a FL/LU/UV-Vis HIDEX (Hidex sense, Hidex, Finland). All steps were performed in the dark to prevent photobleaching.

- 1. *Cytotoxicity test*

HT-22 cells were seeded in a 96-well plate (SPL Life Science, Republic of Korea) containing 200 µL of DMEM-h per well. The cells were then incubated for 24 h to allow for stabilization. After this period, half of the cell media was removed and replaced with 30 µL of 1× PBS, fibril solution, or citric acid-treated fibril solution. The cells were then incubated for an additional 24 h. Subsequently, 10 µL of the CCK-8 solution was added to each well and the absorbance was measured at a wavelength of 450 nm using a SpectraMax ABS Plus microplate reader (Molecular Devices, USA) after 4 h. The number of live and dead cells was determined using a Max-view Live/Dead staining kit (Biomax, Republic of Korea) and images were captured using an LSM700 confocal microscope (Carl Zeiss, Germany). The intensity of confocal fluorescent images was quantified using the ImageJ software.

- 1. *MD simulation*

The interaction between citric acid and α-synuclein fibrils was investigated using Autodock Vina. Before docking, the three-dimensional structures and protonation states of citric acid (PubChem CID 311) and α‑synuclein fibrils (Protein Data Base (PDB) IDs: 6CU7, 6CU8) and monomer (PDB ID: 1XQ8), depending on pH conditions, were optimized using the Avogadro program. Additionally, α-synuclein structures (PDB IDs: 6CU7, 6CU8, 1XQ8) were prepared by removing water molecules, merging non-polar hydrogen atoms, and assigning charges. The binding conformation between citric acid and α-synuclein structures that exhibited the lowest binding energy was selected and subsequently visualized and analyzed using Discovery Studio 2021 and ChimeraX.

1. **Results**
   1. **Preparation of the α-synuclein fibrils**

Before investigating the interaction between citric acid and α-synuclein, we synthesized the α-synuclein fibrils by incubating 1 mg·mL^-1^ of its monomers in PBS with shaking at 1,000 RPM. AFM imaging of α-synuclein fibrils revealed the successful fabrication of amyloid fibrillar aggregates (Fig. S1A). In the AFM image analyzed NX10 software, we observed that the α-synuclein fibrils have 7.13 ± 1.44 nm in height (Fig. S1B). Additionally, the ThT fluorescence intensity of α-synuclein fibrils (1298 ± 204 arbitrary units (a.u.)), compared to that of the control group (DW, 29 ± 8 a.u.), indicates that they have abundant β-sheet conformation of α-synuclein fibrils (Fig. S1C). This successful synthesis and preparation of α-synuclein fibrils enabled further systematic analyses for investigating the interaction between citric acid and α-synuclein fibrils.

- 1. **Citric acid inhibits α-synuclein fibrillization and disassembles α-synuclein fibrils**

To explore the inhibitory effects of citric acid on α-synuclein aggregation, α-synuclein monomers were incubated with 0, 1, and 5 mM citric acid during fibrillization; afterward, ThT assays (Fig. S2) were performed. The results showed that, compared to the 0 mM condition, the relative fluorescence intensity of α-synuclein aggregates decreased by 31.8% and 78.6% for 1 and 5 mM citric acid, respectively. These findings indicate that citric acid can inhibit α-synuclein aggregation and suggest its potential to interrupt β-sheet formation within the aggregates [34].

To assess the effectiveness of citric acid in disassembling α-synuclein fibrils across incubation times, α-synuclein fibrils were reacted with 5 mM of citric acid for three days (Fig. S3). A notable reduction in fibril length was observed with prolonged incubation. Extending the incubation time (one day to three days) with 5 mM of citric acid results in a shift of the average length of α-synuclein fibrils from 0.79 ± 0.55 μm to 0.31 ± 0.21 μm. This result showed that fibrils reacted with 5 mM citric acid for three days decreased by 23.3% compared to the initial length and indicated that reduction of fibril length depends on citric acid concentration and reaction time. These results are consistent with previous studies of small molecules (CNS-11, and CNS-11g) which can effectively disaggregate α-synuclein fibrils [35].

The ability of citric acid to disassemble α-synuclein fibrils was further validated through an immunoblot assay, as shown in Fig. S4. The results showed that the band of α-synuclein fibril almost faded away with 5 mM citric acid compared to that of α-synuclein fibril without citric acid. In addition, the AFM analysis in Fig. S5A shows that disassembled fibril fragments were abundantly observed in citric acid-treated α-synuclein fibrils, compared to the untreated fibril (Fig S1A). All AFM images were quantitively analyzed using the NX10 software and the results showed that the α-synuclein fibrils reduced from 7.13 ± 1.44 nm to 0.53 ± 19 nm in average height when they were treated with 5mM of citric acid (Fig. S5B). These results confirm the ability of citric acid to disassemble α-synuclein fibrils, suggesting that citric acid has the potential to remove fibrillar α-synuclein aggregates in physiological conditions.

We analyzed the conformational change in α-synuclein fibrils with citric acid using the ThT assays in artificial cerebrospinal fluid (aCSF) to investigate how citric acid disassembles α-synuclein fibrils because α-synuclein fibrils exist in the patient’s brain (Fig. S6A). The results showed that the fluorescence intensity of α-synuclein fibrils treated with 5 mM citric acid decreased by 26%, indicating that citric acid can be effective under aCSF conditions. To further investigate the effect of citric acid (10 mM) on α-synuclein fibrils regardless of pH conditions, we adjusted pH with or without KOH (Fig. S6B). Notably, both conditions (e.g., pH 7 and pH 3) resulted in a reduction in ThT fluorescence signal, indicating disassembly of α-synuclein aggregates. Specifically, although to a lesser extent than the acidic condition (~pH 3), the pH-neutralized citric acid still resulted in a 32% reduction in ThT fluorescence intensity. These results demonstrate that citric acid can disaggregate α-synuclein fibrils even at neutral pH. These propensities of citric acid breaking down the β-strand structure of amyloid fibrils are comparable to previous studies of α-synuclein fibril-disassembling small molecules [35].

- 1. **Cytotoxicity of citric acid**

To select the optimal concentration of citric in a cellular assay, we evaluated the cytotoxicity of citric acid at various concentrations using neuronal cell lines (HT-22 and SH-SY5Y), the two commonly used cell models for neurological studies [36, 37] (Fig. S7). We observed that 5 mM and 10 mM of citric acid resulted in a 12.3 and 63.9% reduction in cell viability for HT-22 cells, respectively. Similarly, the viability of SH-SY5Y cells was reduced by 6.1 and 18.5% for 5 and 10 mM of citric acid, respectively. These results revealed that the use of citric acid above 5 mM is cytotoxic to both types of neuronal cells, thereby compromising the therapeutic effects of citric acids on α-synuclein fibrils. Therefore, we selected 5 mM of citric acid for further experiments because this concentration sufficiently affected the conformation of fibrils while minimally impacting neuronal cell viability.

- 1. **Molecular docking simulation**

To simulate the pH-dependent binding behavior of citric acid to α-synuclein fibril (PDB ID: 6CU7), the protonation states of citric acid and relevant amino acid residues were adjusted according to the target pH values (7.2 and 2.9) using Avogadro software and standard pKa reference data. Notably, the acidic condition (pH 2.9) was chosen based on the intrinsic pH of 10 mM citric acid, which measured approximately pH 2.98. At pH 2.9, citric acid formed the strongest and most localized interactions, consisting entirely of conventional hydrogen bonds with LYS45, HIS50, and GLU57, resulting in the highest binding affinity of –4.966 kcal/mol (Fig. S8 and Table S3). Interestingly, despite differences in protonation state and interaction strength, citric acid consistently bound to spatially similar regions of the fibril across pH conditions. These regions are located within or adjacent to the β-sheet-forming segments of α-synuclein fibrils, particularly from β2 to β5 [38]. This suggests a pH-independent binding preference for structural core regions that are critical for fibril integrity. Consequently, citric acid may exert its destabilizing effect by interfering with the hydrogen bonding network and structural order of these β-sheet domains.

To further explore the pH-dependent interactions of citric acid with α-synuclein fibrils, we additionally performed MD simulations using the distinct fibril structure of PDB ID: 6CU8 (Fig. S9 and Table S4). At pH 2.9, citric acid formed multiple conventional hydrogen bonds with THR54, ALA56 and THR72 along with a carbon hydrogen bond to GLY73, although the overall binding affinity slightly decreased to –4.994 kcal/mol. These results indicate that citric acid stably interacts with the 6CU8 fibril through hydrogen bonding at both pH conditions, but with pH-dependent variation in binding mode and residue specificity. Notably, citric acid consistently engaged residues located near β-sheet-forming regions, particularly THR54, THR72, and GLY73, which are associated with β-strands β3 to β5 [38]. This suggests a potential mechanism by which citric acid may weaken fibril integrity by locally disrupting the β-sheet hydrogen bonding network

We also conducted an MD simulation involving citric acid and an α-synuclein monomer to investigate the inhibitory effect of citric acid on α-synuclein fibrillation. In this simulation, the H domain of citric acid formed a conventional hydrogen bond with GLY36, while the O domain engaged in an attractive charge interaction with LYS32, a conventional hydrogen bond with VAL40, and a carbon hydrogen bond with GLY36. These interactions resulted in a binding affinity of –4.077 kcal·mol⁻¹ (Table S5) at pH 7.2. The results show that there exists an interaction between citric acid and the β-strand formation region (VAL40) of α-synuclein monomer, and this interaction provides a theoretical basis for the inhibitory effect on the fibrillization of α-synuclein. Thus, we postulate that citric acid exhibits a prophylactic effect that prevents the formation of α-synuclein fibrils by interacting with an α-synuclein monomer. Both MD results of citric acid on α-synuclein fibril and monomer suggest that citric acid may offer both prophylactic and therapeutic effects by preventing the formation of fibrils and disassembling existing α-synuclein fibrils.

1. **Discussion**

The presence of fibrillar α-synuclein aggregates in the brain of synucleinopathy patients is a crucial factor contributing to pathological progression. These aggregated states of α-synuclein serve as seed sites for further amyloid agglomeration, leading to the spread of soluble α-synuclein oligomers and neurotoxicity [39]. Despite ongoing clinical trials of PD in 2022, approximately 83% of drug candidates primarily focus on symptom relief and slowing disease progression [40]. To date, various approaches have been developed to inhibit α-synuclein aggregation, including small molecules, antibodies, and engineered peptides [16-23]. Although these approaches can effectively prevent additional α-synuclein aggregates, existing local α-synuclein aggregates remain active in the brain and fuel disease progression [24, 25]. Therefore, the most desirable strategy for pathological treatment involves both the inhibition of α-synuclein fibrillization and the elimination of existing fibrillar α-synuclein aggregates in the brain of synucleinopathy patients.

Citric acid is a naturally occurring compound that is particularly abundant in citrus fruits such as oranges and lemons [26, 27]. Compounds found in citrus fruits exhibit neuroprotective effects by reducing oxidative stress levels [28, 31, 41]. Our finding provides further insight into these studies for understanding the neuroprotective effects of citric acid in synucleinopathies. Specifically, we quantitatively revealed that citric acid can disassemble the fibrillar network of α-synuclein aggregates, through a TEM image and AFM analysis. We confirmed that α-synuclein fibril disassembly occurs due to deformation of β-sheet structure caused by citric acid through the FT-IR, CD, and ThT assays. We also revealed that citric acid can inhibit the additional formation of the β-sheet structure of α-synuclein. Through MD analysis, we observed that citric acid exhibits a high affinity for the β-sheet and adjacent regions in both α-synuclein monomers and fibrils. Additionally, our study demonstrated that citric acid alleviates the cytotoxicity of α-synuclein fibrils, resulting in a notable 50% reduction in the cytotoxicity of the neuronal cell models. These findings suggest that citric acid has great potential as a treatment for synucleinopathies by targeting fibrillar α-synuclein aggregates.

While we have demonstrated a novel capability of citric acid, further validation is required to determine its effectiveness *in vivo*. Despite these limitations, our research offers great insight for designing drug candidates that can effectively remove α-synuclein aggregates in synucleinopathies. According to the recent reports [42, 43], α-synuclein aggregates in the enteric nervous system lead to gut dysfunction and propagate to the brain, which triggers brain pathologies, including neurodegeneration and motor symptoms. In this respect, we speculate that citric acid can hinder this mechanism because it has a low molecular weight, allowing it to be easily absorbed into the gastrointestinal system. Therefore, our future study is to observe the effectiveness of a citric acid-containing daily diet in an *in vivo* study for gut-seeded α-synuclein fibrils and also in the brain. Through these processes, we hope that our studies can provide insight into the discovery of fibrillar α-synuclein aggregate-disassembling agents and secure the optimal drug candidates for synucleinopathies.

**References**

[1] P. Calabresi, A. Mechelli, G. Natale, et al., Alpha-synuclein in Parkinson’s disease and other synucleinopathies: from overt neurodegeneration back to early synaptic dysfunction, Cell death & disease 14 (2023) 176.

[2] J. Burré, M. Sharma, T. Tsetsenis, et al., α-Synuclein promotes SNARE-complex assembly *in vivo* and *in vitro*, Science 329 (2010) 1663-1667.

[3] J. Lotharius, P. Brundin, Pathogenesis of Parkinson's disease: dopamine, vesicles and α-synuclein, Nature Reviews Neuroscience 3 (2002) 932-942.

[4] J. Xu, S.-Y. Kao, F.J. Lee, et al., Dopamine-dependent neurotoxicity of α-synuclein: a mechanism for selective neurodegeneration in Parkinson disease, Nature medicine 8 (2002) 600-606.

[5] G.K. Wenning, K.A. Jellinger, The role of α-synuclein in the pathogenesis of multiple system atrophy, Acta neuropathologica 109 (2005) 129-140.

[6] L. Bertram, R.E. Tanzi, The genetic epidemiology of neurodegenerative disease, The Journal of clinical investigation 115 (2005) 1449-1457.

[7] P. Flagmeier, G. Meisl, M. Vendruscolo, et al., Mutations associated with familial Parkinson’s disease alter the initiation and amplification steps of α-synuclein aggregation, Proceedings of the National Academy of Sciences 113 (2016) 10328-10333.

[8] J.Y. Han, T.S. Choi, H.I. Kim, Molecular role of Ca2+ and hard divalent metal cations on accelerated fibrillation and interfibrillar aggregation of α-synuclein, Scientific reports 8 (2018) 1895.

[9] V.N. Uversky, J. Li, A.L. Fink, Pesticides directly accelerate the rate of α-synuclein fibril formation: a possible factor in Parkinson’s disease, FEBS letters 500 (2001) 105-108.

[10] V.N. Uversky, J. Li, K. Bower, et al., Synergistic effects of pesticides and metals on the fibrillation of α-synuclein: implications for Parkinson’s disease, Neurotoxicology 23 (2002) 527-536.

[11] D. Lee, H.G. Jung, D. Park, et al., Biomimetically Engineered Amyloid-Shelled Gold Nanocomplexes for Discovering α-Synuclein Oligomer-Degrading Drugs, ACS applied materials & interfaces 15 (2022) 2538-2551.

[12] V.N. Uversky, J. Li, A.L. Fink, Evidence for a partially folded intermediate in α-synuclein fibril formation, Journal of Biological Chemistry 276 (2001) 10737-10744.

[13] A.K. Buell, C. Galvagnion, R. Gaspar, et al., Solution conditions determine the relative importance of nucleation and growth processes in α-synuclein aggregation, Proceedings of the National Academy of Sciences 111 (2014) 7671-7676.

[14] P. Desplats, H.-J. Lee, E.-J. Bae, et al., Inclusion formation and neuronal cell death through neuron-to-neuron transmission of α-synuclein, Proceedings of the National Academy of Sciences 106 (2009) 13010-13015.

[15] R. Cascella, S.W. Chen, A. Bigi, et al., The release of toxic oligomers from α-synuclein fibrils induces dysfunction in neuronal cells, Nature communications 12 (2021) 1814.

[16] M. Riedel, O. Goldbaum, L. Schwarz, et al., 17-AAG induces cytoplasmic α-synuclein aggregate clearance by induction of autophagy, PloS one 5 (2010) e8753.

[17] J. Pujols, S. Peña-Díaz, D.F. Lázaro, et al., Small molecule inhibits α-synuclein aggregation, disrupts amyloid fibrils, and prevents degeneration of dopaminergic neurons, Proceedings of the National Academy of Sciences 115 (2018) 10481-10486.

[18] Q. Wang, S. Yao, Z.-x. Yang, et al., Pharmacological characterization of the small molecule 03A10 as an inhibitor of α-synuclein aggregation for Parkinson’s disease treatment, Acta pharmacologica Sinica 44 (2023) 1122-1134.

[19] E. Nordström, F. Eriksson, J. Sigvardson, et al., ABBV-0805, a novel antibody selective for soluble aggregated α-synuclein, prolongs lifespan and prevents buildup of α-synuclein pathology in mouse models of Parkinson's disease, Neurobiology of Disease 161 (2021) 105543.

[20] V. Gupta, S. Salim, I. Hmila, et al., Fibrillar form of α-synuclein-specific scFv antibody inhibits α-synuclein seeds induced aggregation and toxicity, Scientific Reports 10 (2020) 8137.

[21] J.H. Torpey, R.M. Meade, R. Mistry, et al., Insights into peptide inhibition of alpha-synuclein aggregation, Frontiers in Neuroscience 14 (2020) 561462.

[22] B. Popova, D. Wang, A. Rajavel, et al., Identification of two novel peptides that inhibit α-synuclein toxicity and aggregation, Frontiers in Molecular Neuroscience 14 (2021) 659926.

[23] B. Zhou, L. Wang, J. Zhang, et al., Inhibition of aggregation and toxicity of α-synuclein in the presence of copper by an N-methylated peptide, Journal of Molecular Structure 1211 (2020) 128079.

[24] S.K. Singh, A. Dutta, G. Modi, α-Synuclein aggregation modulation: an emerging approach for the treatment of Parkinson's disease, Future medicinal chemistry 9 (2017) 1039-1053.

[25] D. Charvin, R. Medori, R.A. Hauser, et al., Therapeutic strategies for Parkinson disease: beyond dopaminergic drugs, Nature Reviews Drug Discovery 17 (2018) 804-822.

[26] X. Yin, J. Li, H.-d. Shin, et al., Metabolic engineering in the biotechnological production of organic acids in the tricarboxylic acid cycle of microorganisms: advances and prospects, Biotechnology advances 33 (2015) 830-841.

[27] S. Nangare, Y. Vispute, R. Tade, et al., Pharmaceutical applications of citric acid, Future Journal of Pharmaceutical Sciences 7 (2021) 1-23.

[28] M. Abdel-SalamOmar, R. YounessEman, A. MohammedNadia, et al., Citric acid effects on brain and liver oxidative stress in lipopolysaccharide-treated mice, Journal of medicinal food (2014).

[29] K. Surapaneni, M. Jainu, Comparative effect of pioglitazone, quercetin and hydroxy citric acid on the status of lipid peroxidation and antioxidants in experimental non-alcoholic steatohepatitis, J Physiol Pharmacol 65 (2014) 67-74.

[30] X. Wu, H. Dai, L. Liu, et al., Citrate reduced oxidative damage in stem cells by regulating cellular redox signaling pathways and represent a potential treatment for oxidative stress-induced diseases, Redox biology 21 (2019) 101057.

[31] O.M. Abdel-Salam, E.R. Youness, N.A. Mohammed, et al., Novel neuroprotective and hepatoprotective effects of citric acid in acute malathion intoxication, Asian Pacific Journal of Tropical Medicine 9 (2016) 1181-1194.

[32] D. Lee, D. Park, I. Kim, et al., Plasmonic nanoparticle amyloid corona for screening Aβ oligomeric aggregate-degrading drugs, Nature communications 12 (2021) 639.

[33] D. Lee, H.G. Jung, D. Park, et al., Bioengineered amyloid peptide for rapid screening of inhibitors against main protease of SARS-CoV-2, Nature Communications 15 (2024) 2108.

[34] M.H. Viet, S.T. Ngo, N.S. Lam, et al., Inhibition of aggregation of amyloid peptides by beta-sheet breaker peptides and their binding affinity, The Journal of Physical Chemistry B 115 (2011) 7433-7446.

[35] K.A. Murray, C.J. Hu, H. Pan, et al., Small molecules disaggregate alpha-synuclein and prevent seeding from patient brain-derived fibrils, Proceedings of the National Academy of Sciences 120 (2023) e2217835120.

[36] Z. Ma, K. Liu, X.-R. Li, et al., Alpha-synuclein is involved in manganese-induced spatial memory and synaptic plasticity impairments via TrkB/Akt/Fyn-mediated phosphorylation of NMDA receptors, Cell death & disease 11 (2020) 834.

[37] Y.-X. Shen, P.-S. Lee, M.-C. Teng, et al., Influence of Cigarette Aerosol in Alpha-Synuclein Oligomerization and Cell Viability in SH-SY5Y: Implications for Parkinson’s Disease, ACS Chemical Neuroscience 15 (2024) 1484-1500.

[38] R. Guerrero-Ferreira, N.M. Taylor, A.-A. Arteni, et al., Two new polymorphic structures of human full-length alpha-synuclein fibrils solved by cryo-electron microscopy, Elife 8 (2019) e48907.

[39] K.C. Luk, C. Song, P. O'Brien, et al., Exogenous α-synuclein fibrils seed the formation of Lewy body-like intracellular inclusions in cultured cells, Proceedings of the National Academy of Sciences 106 (2009) 20051-20056.

[40] K. McFarthing, G. Rafaloff, M. Baptista, et al., Parkinson’s disease drug therapies in the clinical trial pipeline: 2022 update, Journal of Parkinson's Disease 12 (2022) 1073-1082.

[41] K. Liu, X. Wu, H. Dai, Citric acid cross‐linked chitosan for inhibiting oxidative stress after nerve injury, Journal of Biomedical Materials Research Part B: Applied Biomaterials 110 (2022) 2231-2240.

[42] A.J. Bindas, S. Kulkarni, R.A. Koppes, et al., Parkinson's disease and the gut: Models of an emerging relationship, Acta biomaterialia 132 (2021) 325-344.

[43] C. Challis, A. Hori, T.R. Sampson, et al., Gut-seeded α-synuclein fibrils promote gut dysfunction and brain pathology specifically in aged mice, Nature Neuroscience 23 (2020) 327-336.

**Figure captions**


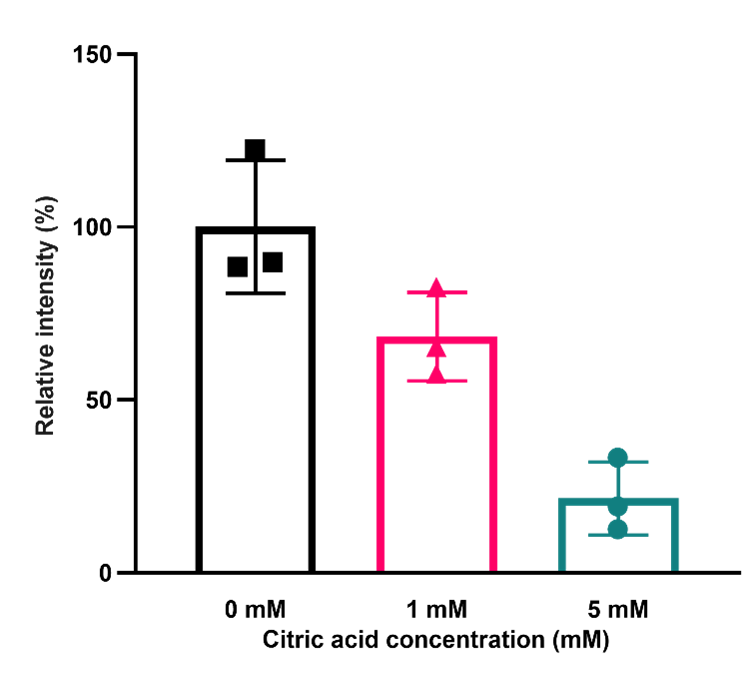


**Fig. S1.** Thioflavin T intensity of α-synuclein monomer solutions incubated with various concentrations of citric acid for five days. The concentration of the monomer solution is 1 mg ·mL^-1^. Each solution is treated with 0 mM (orange), 1 mM (red), and 5 mM (green) of citric acid, respectively. Data are presented as the mean values ± standard deviation from *n* = 3 independent experiments


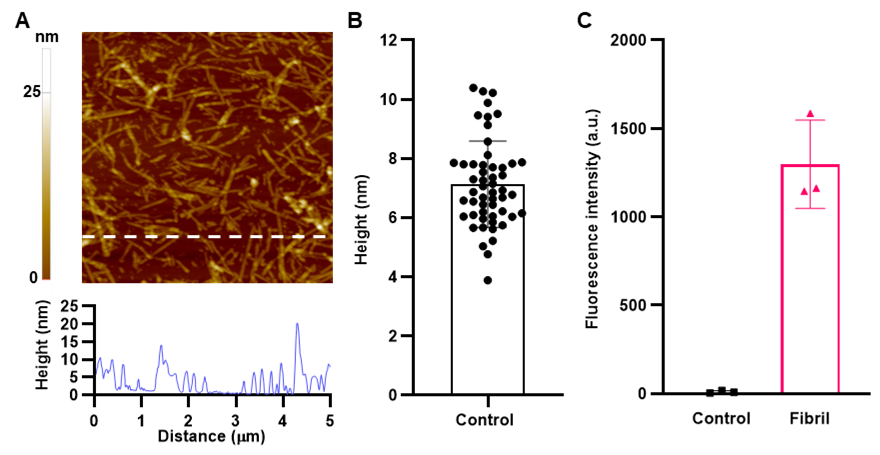


**Fig. S2.** Topological and Thioflavin T (ThT) analysis of α-synuclein fibrils. (A) Topological analysis of α-synuclein fibrils analyzed by atomic force microscopy. The bottom graphs show the topographic cross-sectional profile of a white dotted line. The image is 5 × 5 μm in size. (B) Height of α-synuclein fibrils analyzed by NX10 software. Data are presented as the mean values ± standard deviation from *n* = 53. (C) ThT fluorescence intensity of α-synuclein fibrils and the control group (distilled water). The ThT fluorescence intensities are measured by excitation at 405 nm and emission at 510 nm in wavelength. Data are presented as the mean values ± standard deviation from *n* = 3 independent experiments


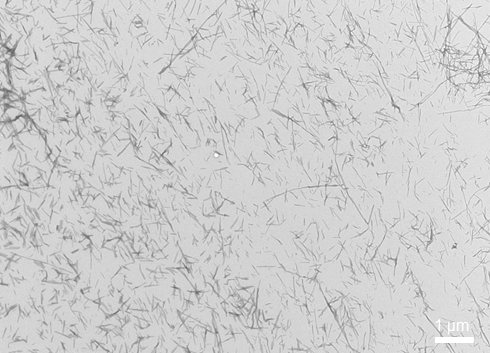


**Fig. S3.** Transmission electron microscopy analysis of α -synuclein fibrils with citric acid treatment. α-synuclein fibrils were reacted with 5 mM of citric acid for three days.


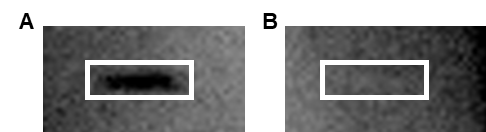


**Fig. S4.** Immunoblotting image of α-synuclein fibrils. α-synuclein fibrils treated with (A) 0 mM of citric acid (non-treated) and (B) 5 mM of citric acid for three days.


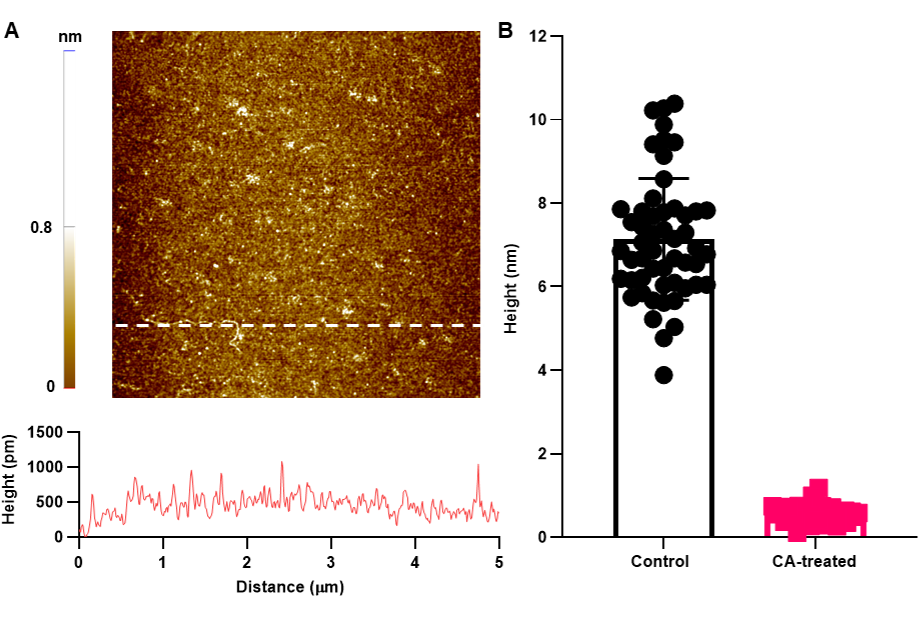


**Fig. S5.** Height analysis of citric acid-treated α-synuclein fibrils analyzed by atomic force microscopy. (A) The topological analysis of citric acid-treated α-synuclein fibrils. The bottom graphs show the cross-sectional profile of a white dotted line. (B) Height of α-synuclein fibrils (control, Fig. S2A) and citric acid treated α-synuclein fibrils. Data are presented as the mean values ± standard deviation from *n* = 53 for control and *n* = 50 for CA-treated fibrils. CA: citric acid.

The concentration of citric acid, reacted with fibrils, is 5 mM and the height of each sample is measured using the NX10 software (Park Systems, Korea). The average height of α-synuclein fibrils and citric acid-treated fibrils are 7.13 ± 1.44 nm and 0.53 ± 0.19 nm, respectively.


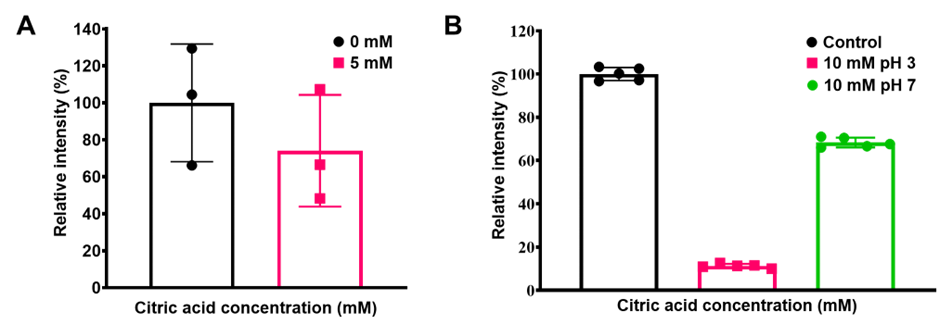


**Fig. S6.** (A) Thioflavin T fluorescence intensity of α-synuclein fibrils treated with citric acid in artificial cerebrospinal fluid (aCSF). α-synuclein fibrils were incubated with 0 mM (black) or 5 mM (red) citric acid in aCSF. (B) Effect of citric acid on α-synuclein fibril disassembly under different pH conditions. A 10 mM citric acid solution (initial pH ~ 3) was adjusted to pH 7 using KOH. α-synuclein fibrils were treated with 0 mM (black), 10 mM citric acid at pH 3 (red), and 10 mM citric acid at pH 7 (green). Data represent mean ± standard deviation from *n* ≥ 3 independent experiments.


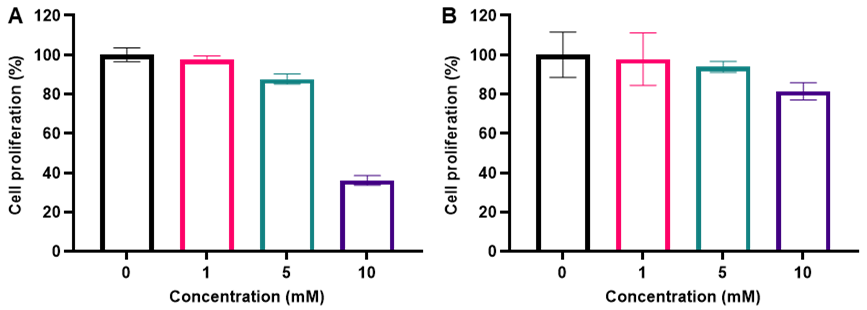


**Fig. S7.** Cytotoxicity of citric acid-treated in HT-22 cells (A) and SH-SY5Y cells (B). Cells are incubated with 0 mM (orange), 1 mM (red), 5 mM (green), and 10 mM of citric acid (blue). The incubation time of citric acid and SH-SY5Y cells is 24 h. Data are presented as the mean values ± standard deviation from *n* = 3 independent experiments


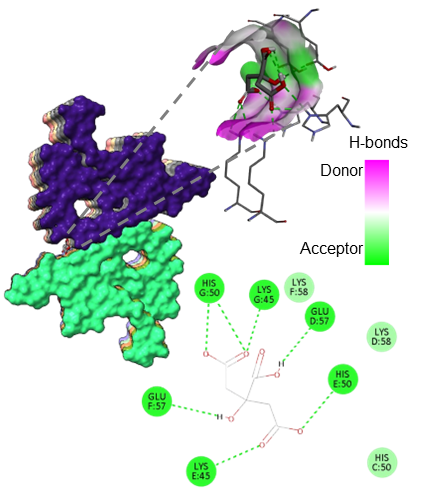


**Fig. S8.** Molecular docking simulation (Autodock Vina) of α-synuclein fibrils (Protein Data Base ID: 6CU7) and citric acid (PubChem CID: 311) at pH 2.9. Surface representations show the fibril structure (purple and green) with the binding site of citric acid highlighted. Insets display the donor–acceptor surface maps, along with 2D interaction diagrams depicting hydrogen bonds (green dashed lines).


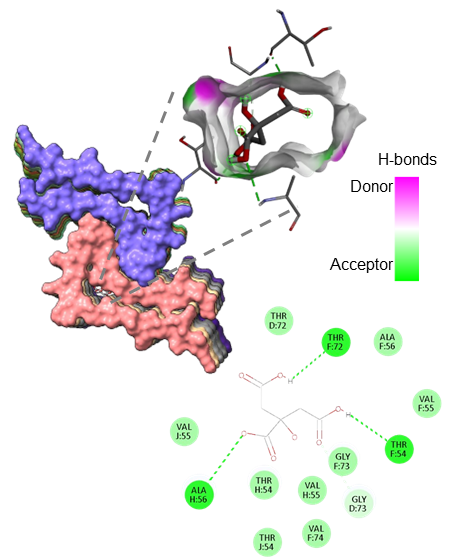


**Fig. S9.** Molecular docking simulation (Autodock Vina) of α-synuclein fibrils (Protein Data Base ID: 6CU8) and citric acid (PubChem CID: 311) at pH 2.9. Surface representations show the fibril structure (purple and green) with the binding site of citric acid highlighted. Insets display the donor–acceptor surface maps, along with 2D interaction diagrams depicting hydrogen bonds (green dashed lines).

**Tables**

**Table S1.** Molecular docking simulation results of citric acid and rod polymorph α-synuclein fibril (Protein Data Base ID: 6CU7) at pH 7.2. LYS; lysine, HZ; ζ-hydrogen, NZ; ζ-nitrogen, HIS; histidine, HD; δ-hydrogen

| Interaction | Distance (Å) | Type | From | To |
| --- | --- | --- | --- | --- |
| F:LYS45:HZ1 - d:Citric_acid1:O | 2.45438 | Salt Bridge;Attractive Charge | F:LYS45:HZ1 | d:Citric_acid1:O |
| F:LYS45:HZ2 - d:Citric_acid1:O | 2.77531 | Salt Bridge;Attractive Charge | F:LYS45:HZ2 | d:Citric_acid1:O |
| F:LYS45:HZ3 - d:Citric_acid1:O | 2.57745 | Salt Bridge;Attractive Charge | F:LYS45:HZ3 | d:Citric_acid1:O |
| H:LYS45:HZ1 - d:Citric_acid1:O | 2.44099 | Salt Bridge;Attractive Charge | H:LYS45:HZ1 | d:Citric_acid1:O |
| D:LYS45:NZ - d:Citric_acid1:O | 4.6402 | Attractive Charge | D:LYS45:NZ | d:Citric_acid1:O |
| F:HIS50:HD1 - d:Citric_acid1:O | 2.36038 | Conventional Hydrogen Bond | F:HIS50:HD1 | d:Citric_acid1:O |
| H:LYS45:HZ2 - d:Citric_acid1:O | 2.77802 | Conventional Hydrogen Bond | H:LYS45:HZ2 | d:Citric_acid1:O |
| d:Citric_acid1:O - D:HIS50 | 4.13643 | Pi-Anion | d:Citric_acid1:O | D:HIS50 |

**Table S2.** Molecular docking simulation results of citric acid and twist polymorph α-synuclein fibril (Protein Data Base ID: 6CU8) at pH 7.2. THR; threonine, HG; γ- hydrogen, GLY; glycine, CA; α-carbon

| Interaction | Distance (Å) | Type | From | To |
| --- | --- | --- | --- | --- |
| G:THR59:HG1 - d:Citric_acid1:O | 2.56861 | Conventional Hydrogen Bond | G:THR59:HG1 | d:Citric_acid1:O |
| d:Citric_acid1:H - E:THR72:O | 3.08784 | Conventional Hydrogen Bond | d:Citric_acid1:H | E:THR72:O |
| C:GLY73:CA - d:Citric_acid1:O | 3.72641 | Carbon Hydrogen Bond | C:GLY73:CA | d:Citric_acid1:O |

**Table S3**. Molecular docking simulation results of citric acid and rod polymorph α-synuclein fibril (Protein Data Base ID: 6CU7) at pH 2.9. GLU; glutamate, OE; ε-oxygen, LYS; lysine, HZ; hydrogen on ζ-nitrogen, HIS; histidine, HD; δ-hydrogen

| Interaction | Distance (Å) | Type | From | To |
| --- | --- | --- | --- | --- |
| d:Citric_acid1:H - F:GLU57:OE1 | 2.69609 | Conventional Hydrogen Bond | d:Citric_acid1:H | F:GLU57:OE1 |
| d:Citric_acid1:H - D:GLU57:OE1 | 2.47565 | Conventional Hydrogen Bond | d:Citric_acid1:H | D:GLU57:OE1 |
| E:LYS45:HZ1 - d:Citric_acid1:O | 2.77715 | Conventional Hydrogen Bond | E:LYS45:HZ1 | d:Citric_acid1:O |
| E:LYS45:HZ2 - d:Citric_acid1:O | 2.758 | Conventional Hydrogen Bond | E:LYS45:HZ2 | d:Citric_acid1:O |
| E:HIS50:HD1 - d:Citric_acid1:O | 2.30941 | Conventional Hydrogen Bond | E:HIS50:HD1 | d:Citric_acid1:O |
| G:LYS45:HZ2 - d:Citric_acid1:O | 2.23228 | Conventional Hydrogen Bond | G:LYS45:HZ2 | d:Citric_acid1:O |
| G:HIS50:HD1 - d:Citric_acid1:O | 2.8032 | Conventional Hydrogen Bond | G:HIS50:HD1 | d:Citric_acid1:O |
| G:HIS50:HD1 - d:Citric_acid1:O | 2.79781 | Conventional Hydrogen Bond | G:HIS50:HD1 | d:Citric_acid1:O |

**Table S4.** Molecular docking simulation results of citric acid and twist polymorph α-synuclein fibril (Protein Data Base ID: 6CU8) at pH 2.9. THR; threonine, ALA; alanine, HN; back bone amine hydrogen, GLY; glycine, CA; α-carbon

| Interaction | Distance (Å) | Type | From | To |
| --- | --- | --- | --- | --- |
| d:Citric_acid1:H - F:THR72:O | 2.08234 | Conventional Hydrogen Bond | d:Citric_acid1:H | F:THR72:O |
| d:Citric_acid1:H - F:THR54:O | 2.38567 | Conventional Hydrogen Bond | d:Citric_acid1:H | F:THR54:O |
| H:ALA56:HN - d:Citric_acid1:O | 3.09076 | Conventional Hydrogen Bond | H:ALA56:HN | d:Citric_acid1:O |
| D:GLY73:CA - d:Citric_acid1:O | 3.55061 | Carbon Hydrogen Bond | D:GLY73:CA | d:Citric_acid1:O |

**Table S5.** Molecular docking simulation results of citric acid and α-synuclein monomer (Protein Data Base ID: 1XQ8) at pH 7.2. LYS; lysine, NZ; ζ-nitrogen, GLY; glycine, VAL; valine, HN; back bone amine hydrogen, CA; α-carbon

| Interaction | Distance (Å) | Type | H-Donor | H-Acceptor |
| --- | --- | --- | --- | --- |
| A:LYS32:NZ - d:Citric_acid1:O | 4.56124 | Attractive Charge | A:LYS32:NZ | d:Citric_acid1:O |
| d:Citric_acid1:H - A:GLY36:O | 2.45445 | Conventional Hydrogen Bond | d:Citric_acid1:H | A:GLY36:O |
| A:VAL40:HN - d:Citric_acid1:O | 2.05092 | Conventional Hydrogen Bond | A:VAL40:HN | d:Citric_acid1:O |
| A:GLY36:CA - d:Citric_acid1:O | 3.49374 | Carbon Hydrogen Bond | A:GLY36:Citric acid | d:Citric_acid1:O |
